# A Review on the Application of Magnetic Nanomaterials for Environmental and Ecological Remediation

**DOI:** 10.3390/toxics13100814

**Published:** 2025-09-25

**Authors:** Nan Lu, Yingying Sun, Yan Li, Zhe Liu, Na Wang, Tingting Meng, Yuhu Luo

**Affiliations:** 1 Technology Innovation Center for Land Engineering and Human Settlements, Shaanxi Provincial Land Engineering Construction Group Co., Ltd., Xi’an Jiaotong University, Xi’an 712000, China; sunyy@chd.edu.cn; 2 Key Laboratory of Degraded and Unused Land Consolidation Engineering, Ministry of Natural Resources, Xi’an 710075, China; liyan_hhu@163.com (Y.L.); liuzhe168@126.com (Z.L.); na0113@126.com (N.W.); tt13636712545@163.com (T.M.); huyu_luo@163.com (Y.L.); 3 Institute of Land Engineering and Technology, Shaanxi Provincial Land Engineering Construction Group Co., Ltd., Xi’an 710075, China; 4 Shaanxi Agricultural Development Group Co., Ltd., Xi’an 710075, China

**Keywords:** magnetic nanomaterials, wastewater treatment, soil remediation, heavy metal contamination, organic pollutants, biocompatibility

## Abstract

Despite the immense potential in environmental remediation, the translation of magnetic nanomaterials (MNMs) from laboratory innovations to practical, field-scale applications remains hindered by significant technical and environmental challenges. This is particularly evident in soil environments—which are inherently more complex than aquatic systems and have received comparatively less research attention. Beginning with an outline of the fundamental properties that make iron-based MNMs effective as adsorbents and catalysts for heavy metals and organic pollutants, this review systematically examines their core contaminant removal mechanisms. These include adsorption, catalytic degradation (e.g., via Fenton-like reactions), and magnetic recovery. However, the practical implementation of MNMs is constrained by several key limitations, such as particle agglomeration, oxidative instability, and reduced efficacy in multi-pollutant systems. More critically, major uncertainties persist regarding their long-term environmental fate and biocompatibility. In light of these challenges, we propose that future efforts should prioritize the rational design of stable, selective, and intelligent MNMs through advanced surface engineering and interdisciplinary collaboration.

## 1. Introduction

The global ecological environment is facing unprecedented challenges, driven by rapid industrialisation and continuous population growth. The widespread contamination of water and soil systems by heavy metals and organic pollutants poses a severe threat to ecosystem integrity and human health [1,2], making the development of effective remediation strategies an urgent priority. In this context, environmental geochemistry and health risk assessments often classify elements into two major categories: essential trace elements and potentially toxic elements (PTEs). Essential trace elements, such as Fe, Cu, Zn, Mn, Mo, Se and Cr (III), play critical roles in biological processes, including electron transfer, enzyme catalysis and maintaining redox balance. In contrast, PTEs, such as cadmium (Cd), lead (Pb), mercury (Hg), arsenic (As), chromium (VI) (Cr(VI)), antimony (Sb), and nickel (Ni), have no known beneficial metabolic functions and can exert toxic effects even at very low concentrations [3,4,5,6].

Although conventional remediation strategies, such as chemical precipitation, oxidation processes and biological treatments, have achieved certain outcomes, they remain limited by inherent drawbacks including moderate efficiency, high operational costs and the potential for secondary contamination [7]. These limitations prevent them from being widely applied in real-world scenarios.

Nanotechnology has introduced innovative solutions to these challenges. In particular, adsorption-based approaches have gained attention due to their operational simplicity and potential for high efficiency. However, the performance of these technologies relies heavily on the development of advanced adsorbents. Nanoscale materials, with their high surface area-to-volume ratios and tunable surface chemistry, offer significant advantages for capturing and removing pollutants [8,9]. Recent reviews have further systematized the design principles of nanoadsorbents for heavy metal removal [10]. However, the practical application of conventional nanomaterials is often limited by the difficulty of separating and recovering them after use, which can lead to secondary pollution and high economic costs.

Magnetic nanomaterials (MNMs), particularly iron-based oxides such as Fe_3_O_4_ and γ-Fe_2_O_3_, have emerged as a promising solution to these recovery challenges. Their unique superparamagnetic properties enable them to be separated efficiently from treated media via external magnetic fields, overcoming a critical barrier in nanoparticle-based remediation [11]. This feature, along with their applicability in both environmental analysis and remediation, has positioned MNMs as a versatile tool in environmental technology [12]. Furthermore, MNMs can be easily modified with organic polymers (e.g., chitosan or polyethyleneimine) or inorganic coatings (e.g., silica or graphene oxide), thereby improving their stability, selectivity and adsorption capacity towards target pollutants such as heavy metals and organic dyes [13,14,15]. The strategic surface engineering of iron oxide nanoparticles specifically for environmental remediation has been extensively discussed. For instance, Wang et al. [16] demonstrated that MnFe_2_O_4_–biochar composites could achieve removal rates exceeding 90% for antimony (III) and 85% for cadmium (II) in aqueous solutions. Similarly, magnetic hemicellulose-based composite microspheres incorporating Fe_3_O_4_ nanoparticles showed enhanced adsorption capacity for copper ions [17], and chitosan-coated MNMs particles reached an adsorption capacity of up to 149.25 mg/g for Cu (II) [18]. For heavy metal remediation, the amino-functionalized magnetic cobalt ferrite chitosan beads (NH_2_-CF-CB) exhibited a high adsorption capacity of 158.73 mg/g for Cu (II) ions [19].

Beyond the remarkable adsorption of heavy metals, MNMs have also demonstrated exceptional capability in the degradation and mineralization of refractory organic pollutants [20]. This is primarily achieved through their role as efficient catalysts in Advanced Oxidation Processes (AOPs), such as Fenton-like reactions and peroxymonosulfate (PMS) activation, generating highly reactive oxygen species (ROS) like hydroxyl radicals (•OH) and sulfate radicals (SO_4_•^−^) [21,22]. For instance, multifunctional magnetic biochar (MMBC-400), derived from the co-pyrolysis of corn cob and red mud, can integrate adsorption with catalytic degradation. Such materials not only adsorb malachite green (MG) with high efficiency (up to 793.51 mg/g) but also activate peroxydisulfate (PDS) to generate ROS, achieving a degradation rate exceeding 85% through combined radical (•OH, SO_4_•^−^) and non-radical (^1^O_2_, electron transfer) pathways. This dual functionality of adsorption-degradation synergy positions MNMs as versatile platforms for tackling complex organic contaminants, surpassing the mere phase transfer achieved by traditional adsorbents [23]. For organic pollutants, the same NH_2_-CF-CB adsorbent achieved an exceptional capacity of 357.16 mg/g for malachite green dye, demonstrating its versatile effectiveness [19].

**Figure 1:**
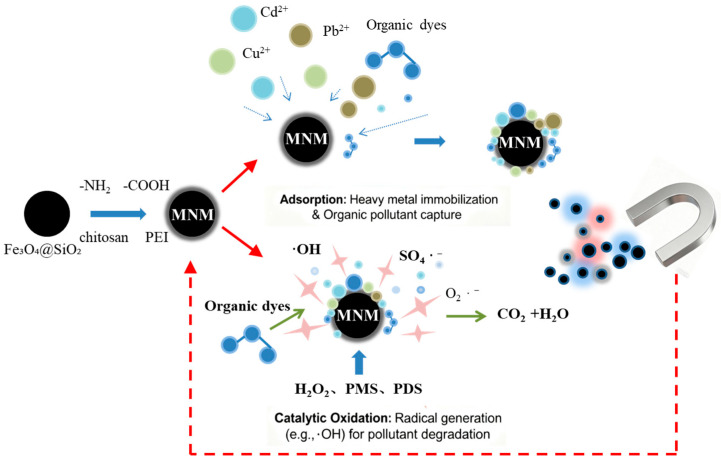
Schematic illustration of the design, functionalization, and application of magnetic nanomaterials (MNMs) for environmental remediation.

A general overview of the functionalization and application mechanisms of MNMs is illustrated in Figure 1.

Magnetic nanomaterials (MNMs) possess a unique set of physicochemical properties that make them highly suitable for environmental remediation. These core properties, along with their primary functions and representative applications, are summarized in Table 1.

Previous reviews have often discussed magnetic nanomaterials (MNMs) within broader contexts of nanotechnology or green chemistry, with a primary focus on their adsorption or catalytic performance. However, fewer efforts have been made to systematically analyze the practical challenges that limit their field-scale application, such as material aggregation, instability, and reduced effectiveness [24] in complex soil environments. This review seeks to extend the current discussion by particularly examining the dual-functionality of MNMs (adsorption-degradation synergy) and their application in soil remediation, an area that has received less attention compared to water treatment. Additionally, we incorporate a dedicated discussion on the biocompatibility and environmental safety of MNMs, as we consider these factors critical for assessing their overall suitability in remediation scenarios. By synthesizing recent advances and ongoing challenges, this review aims to provide a practical overview of the current state of MNMs and outline potential pathways for developing more effective and environmentally sustainable remediation strategies.

## 2. Methodology for Literature Selection

**Figure 2:**
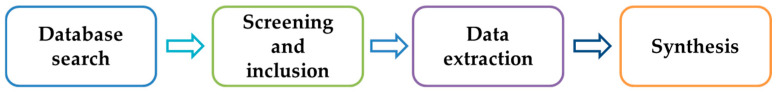
Diagram of the workflow.

The literature search and selection process for this review was conducted in a systematic manner to ensure comprehensiveness, reproducibility, and minimal bias. The methodology primarily involved four stages: (1) database search; (2) screening and inclusion; (3) data extraction; and (4) synthesis. The overall workflow is schematically summarized in Figure 2.

### 2.1. Search Strategy

To identify all relevant publications, a comprehensive electronic literature search was performed using major scientific databases, including Web of Science Core Collection, Scopus, PubMed, and Google Scholar. The search was limited to articles published in English between September 2016 and August 2025. The search strategy employed a combination of keywords and Boolean operators (AND, OR) related to the core concepts of this review. The primary search terms included the following: (“magnetic nanomaterials” OR “Fe_3_O_4_ nanoparticles” OR “superparamagnetic iron oxide”) AND (“adsorption” OR “degradation” OR “Fenton-like” OR “photocatalysis”) AND (“heavy metal” OR “organic pollutant”) AND (“wastewater” OR “soil remediation”) AND “biocompatibility”.

### 2.2. Inclusion and Exclusion Criteria

The inclusion criteria were designed to select original research articles that contribute directly to our understanding of MNMs in environmental remediation. Specifically, we included peer-reviewed journal publications focusing on the synthesis, modification and application of MNMs for removing pollutants, provided they reported essential quantitative data such as removal efficiency, adsorption capacity, catalytic performance, or comprehensive material characterization.

We excluded non-primary sources such as conference abstracts, editorials, and patents. Furthermore, studies not published in English, those with inaccessible full text, and articles that merely utilized commercial magnetic materials without introducing novel modifications or substantive application insights were also omitted to ensure the relevance and novelty of the analyzed literature.

### 2.3. Literature Screening

Initially, all retrieved records were imported into reference management software. After removing duplicates, the titles and abstracts of the remaining articles were screened against the inclusion and exclusion criteria. Potentially relevant articles were then subjected to a full-text review to determine their final eligibility. Any discrepancies during the screening process were resolved through discussion between the authors until a consensus was reached.

### 2.4. Data Extraction and Synthesis

Key data from the included studies was extracted and entered into a table. The extracted information included as follows: (1) the first author and publication year; (2) the type of magnetic nanomaterial; (3) the target pollutant; (4) the experimental conditions; (5) the key performance indicators (removal efficiency and adsorption capacity); (6) the main mechanisms; and (7) the reusability data. The synthesised data were analysed thematically to identify current research trends, technological advances, areas of consensus and existing challenges in the field.

## 3. Applications and Research Progress in Water Pollution Control

The presence of heavy metals and organic pollutants in water poses a substantial challenge in the context of global environmental governance. Statistical analysis indicates that over 70% of global water pollution incidents are attributable to heavy metal pollution, with Pb (II), Cd (II), and Hg (II) identified as priority pollutants due to their high toxicity and bioaccumulation potential [25]. It is estimated that approximately 85% of the water in the Yamuna River, the largest tributary of the Ganges River in India, is classified as highly contaminated with heavy metals [26,27]. The primary heavy metal elements that are directly discharged into the sea include Cr (VI), Pb, Hg, and Cd. Concurrently, organic pollutants, including dyes, pesticides, and pharmaceutical residues, enter water bodies through industrial emissions and agricultural runoff. The recalcitrance of these pollutants, evidenced by their resistance to degradation and ecological toxicity, serves only to exacerbate the water environment crisis [28]. This phenomenon poses a number of threats, both detrimental to ecosystems and posing a threat to the health of human beings. MNMs have been demonstrated to exhibit significant advantages in the field of water pollution control, owing to their rapid separation characteristics and multifunctional surface design [29].

### 3.1. Removal of Heavy Metal Ions

MNMs have garnered significant attention for their ability to eliminate heavy metal pollutants through a combination of mechanisms, including adsorption (governed by physical/chemical interactions), reduction (via electron transfer), and ion exchange (facilitated by surface functional group coordination). A notable example is nano-zero-valent iron (nZVI). Tarekegn et al. demonstrated that nZVI-supported biochar could remove over 99% of Cd within 40 min [30]. However, a critical limitation of nZVI is its susceptibility to oxidation and subsequent deactivation, which restricts its long-term efficacy in practical applications [31]. This inherent trade-off between high reactivity and poor environmental stability represents a central challenge for the field application of many reactive MNMs.

#### 3.1.1. Surface Functionalization and Composites

To overcome the limitations of single-component MNMs like nZVI, researchers have developed composite and structurally modified materials. A prevalent strategy is surface functionalization. A widely adopted strategy involves the introduction of specific functional groups onto MNM surfaces. For example, functionalization with amino (–NH_2_) or carboxyl (–COOH) groups has been shown to markedly enhance adsorption capacity and selectivity toward target pollutants. Lisandra et al. [32] synthesized Fe_3_O_4_ nanoparticles via co-precipitation and cross-linked them into spherical MAAC composites for cadmium removal. The optimal adsorption performance of the prepared MAAC composite for Cd (II) was observed at pH 6. This pH-dependent behavior is consistent with the protonation/deprotonation process of functional groups on the material surface: at pH 6, these groups become fully deprotonated and negatively charged, facilitating the electrostatic attraction toward positively charged Cd (II). This strongly demonstrates that the adsorption is primarily governed by the functional layers introduced via modification, rather than by the magnetic core. Similarly, Cheng et al. [33] prepared amino-functionalized core–shell magnetic nanoparticles (Fe_2_O_3_@mSiO_2_), which exhibited enhanced adsorption for ions like ferric iron (Fe^3+^). Lei et al. [34] modified magnetic iron oxide NPs with 2,3-dimercaptosuccinic acid and dopamine, significantly boosting their adsorption capacity for various heavy metals and demonstrating good reusability.

Beyond organic functionalization, coating MNMs with inorganic oxides or integrating them with conventional adsorbents has also proven highly effective. Xiao et al. [35] combined cerium dioxide nanoparticles with activated carbon to enhance the composite’s adsorption performance. In a cost-effective approach, one study reported the use of volcanic rock coated with α-Fe_2_O_3_ nanoparticles for Cd (II) removal, finding that the nanocoating substantially enhanced adsorption capacity, offering a cost-effective solution [36].

#### 3.1.2. Advanced Composites and Performance

The synthesis of sophisticated multifunctional composites continues to advance the field. One study demonstrated that a magnetic graphene oxide-chitosan composite could adsorb Hg (II) with a remarkable capacity of 339.82 mg/g within just eight minutes. Mechanistic analysis revealed that amidation between chitosan’s amine groups and GO’s hydroxyl groups formed –C(=O)NH– linkages, which were instrumental in achieving superior performance [37]. In another innovative study, the optimal removal efficiency of copper (II) ions by the newly developed chitosan-based nanocomposite film incorporated with 10% *N*-nicotinyl-*N*′, *N*′-bis(pyrrolidinyl) phosphoric triamide and 5% Fe_3_O_4_ nanoparticles (NPs) was achieved at pH 9, reaching 89.44% from a 100 ppm Cu(NO_3_)_2_ solution [38]. This pH-dependent performance can be attributed to the surface charge modulation of functional groups present in the composite. At alkaline pH, the functional groups (such as amino and phosphoric triamide motifs) are largely deprotonated, enhancing electrostatic attraction toward positively charged Cu (II) ions. The incorporation of starch-capped core–shell Fe_3_O_4_ NPs significantly improved the structural integrity and possibly the dispersion of the composite, while the adsorption function was dominated by the chemically functionalized matrix rather than the magnetic core itself.

In summary, the studies above demonstrate that through strategic design—such as grafting specific functional groups or constructing composites—MNMs can achieve high removal efficiency and rapid kinetics for heavy metals. However, it is important to note that these promising results, primarily obtained under optimized laboratory conditions, may not fully represent performance in real wastewater systems characterized by complex compositions and competing ions.

### 3.2. Organic Pollutant Degradation

MNMs have proven highly efficient in degrading organic pollutants through synergistic mechanisms that combine adsorption with catalytic oxidation or reduction. Key degradation pathways include photocatalysis, Fenton-like reactions, and other advanced oxidation processes (AOPs). A significant advantage of MNMs is their rapid recovery via external magnetic fields, which minimizes material loss and reduces operational costs [39]. This section reviews recent advances and persistent challenges in this field.

#### 3.2.1. Mechanisms and Enhancement Strategies

The degradation efficiency of MNMs is largely governed by their ability to generate reactive oxygen species (ROS), such as hydroxyl radicals (•OH) and superoxide anions (•O_2_^−^), which non-selectively oxidize organic pollutants. Strategies to enhance ROS generation include forming heterojunctions to improve charge separation, doping elements to expand light absorption, and constructing composites to leverage synergistic effects.

For instance, Mphuthi et al. [40] synthesized bimetallic zeolitic imidazolate framework (ZIP) nanoparticles, nZIF-8(Zn/Fe) and nZIF-67(Co/Fe), using iron acetylacetonate (Fe(acac)_2_) as a source. The nZIF-67(Co/Fe) variant demonstrated exceptional photocatalytic activity, achieving over 95% decolorization of Rhodamine B (RhB) under illumination while retaining over 90% activity after three cycles, underscoring its stability and reusability.

Doping is another effective approach to enhance photocatalytic performance. Iron (Fe) doping, for example, significantly boosts the activity of graphitic carbon nitride (g-C_3_N_4_). Under optimal conditions (specific Fe concentration, catalyst dosage, and H_2_O_2_ level), Fe-doped g-C_3_N_4_ outperforms its pure form in degrading dyes like RhB. The doped Fe facilitates the separation of photo-generated electron–hole pairs and promotes the generation of •OH and •O_2_^−^, with •OH playing a more dominant role in pollutant degradation [41].

#### 3.2.2. Performance of Fenton-like and Heterogeneous Systems

Fenton-like systems, particularly those activated by visible light, are highly effective for degrading refractory organic pollutants. Munsi et al. [42] synthesised Fe_3_O_4_@DABA (3,5-diaminobenzoic acid-functionalised Fe_3_O_4_ nanoparticles), which exhibited a high adsorption affinity for anionic azo dyes. Maximum adsorption capacities of 259 mg g^−1^ for Congo red and 282 mg g^−1^ for eosin yellow were achieved at pH 3 and 25 °C. The adsorbent demonstrated excellent magnetic recyclability, retaining over 90% of its initial adsorption efficiency after five consecutive cycles. In another study, a magnetic Cu_0.5_Mn_0.5_Fe_2_O_4_ (CMF) catalyst achieved over 99% degradation of RhB (20 mg/L) under visible light in 60 min and maintained a 96% removal rate after five cycles, demonstrating remarkable durability [43]. Similar results were confirmed by Zhu et al. [44], who developed a Fenton-like catalyst based on carboxylcellulose hydrogel-confined Fe_3_O_4_ nanoparticles, which exhibited high catalytic activity and stability for Rhodamine B (RhB) degradation. The high performance is attributed to a synergistic “adsorption–catalysis” mechanism: the hydrogel matrix enriches both RhB and H_2_O_2_ around the Fe_3_O_4_ sites, facilitating efficient •OH generation through the Fe^2+^/Fe^3+^ redox cycle.

### 3.3. Summary

The performance of representative MNMs for removing various pollutants is systematically compared in Table 2, which provides a basis for discussing the prevailing technical challenges and future directions.

Despite the remarkable laboratory performance of MNMs in removing both heavy metals and organic pollutants, their translation into robust, field-ready technologies is impeded by a series of interconnected and fundamental challenges. This section synthesizes the central barriers identified across both research domains to provide a unified perspective on the current limitations and essential future directions.

#### 3.3.1. Material-Centric Limitations

Despite their promising functionalities, the inherent properties of MNMs impose critical constraints on their practical, sustained application. A primary challenge lies in their structural and chemical instability. For example, key materials such as nZVI are highly susceptible to rapid oxidation and deactivation when exposed to air and water [31]. Similarly, although advanced materials such as metal–organic frameworks (MOFs) are highly porous and can be tailored, they often have inferior chemical and thermal stability compared to conventional adsorbents, and their frameworks are prone to collapse in aqueous environments [45]. Furthermore, the complexity and scalability of synthesising these materials present another major barrier. Many high-performance MNMs, including certain MOFs and sophisticated composites, require intricate, expensive and time-consuming synthesis procedures, severely hindering their cost-effective large-scale production [45,46].

#### 3.3.2. Performance and Mechanistic Challenges

Beyond fundamental material stability, MNMs face significant performance and mechanistic challenges in real-world applications. Adequate efficacy and a comprehensive mechanistic understanding under environmentally relevant conditions remain insufficient [47]. A major operational hurdle is regeneration inefficiency and fouling; the adsorption capacity of MNMs can decrease by over 25% after just a few cycles [48,49], as strongly adsorbed pollutants or degradation byproducts foul active surfaces, complicating regeneration and risking secondary pollution [45,50]. Furthermore, the removal mechanisms for complex pollutant mixtures are often ambiguous. In some cases, strong adsorption can masquerade as catalytic performance, merely transferring pollutants from water to the solid phase instead of degrading them [50]. Finally, a critical constraint for photocatalytic MNMs is light dependency, which severely limits their all-weather, around-the-clock operation and practicality in turbid or sub-surface water sources.

#### 3.3.3. Environmental and System Complexity

Real-world wastewater systems introduce complexities rarely encountered in laboratory studies, primarily stemming from complex matrix effects and the challenge of multi-pollutant systems. The presence of co-existing ions, natural organic matter, and diverse pollutants in authentic wastewater can compete for active sites on MNMs, scavenge reactive oxygen species (ROS), or otherwise interfere with the targeted removal process, thereby drastically reducing treatment efficiency [47,50]. Furthermore, research on the synergistic removal mechanisms for composite pollution mixtures remains severely inadequate. The performance of MNMs in such complex environments is consequently difficult to predict and is often sub-optimal, highlighting a significant gap between controlled laboratory conditions and actual application scenarios [46].

In conclusion, while strategic material design—through doping, functionalization, and composite formation—has unlocked exceptionally high removal efficiencies and rapid kinetics in the laboratory, these successes are overwhelmingly demonstrated under optimized, simplified conditions. The path from laboratory proof-of-concept to field-scale application is obstructed by challenges related to material stability, regeneration capability, performance in complex matrices, and a lack of deep mechanistic understanding in real environments. Addressing these gaps requires a concerted focus on designing MNMs for durability and ease of regeneration, conducting long-term studies in real wastewater, and developing a fundamental understanding of interactions in multi-pollutant systems. Overcoming these barriers is imperative to fulfilling the potential of MNMs as high-performance, practical solutions for environmental remediation.

## 4. Applications and Research Progress in Soil Pollution Remediation

Soil is a fundamental and complex component of terrestrial ecosystems, and its remediation presents formidable challenges distinct from those in aqueous environments. These challenges stem from the inherent heterogeneity of the soil matrix, the strong sequestration of pollutants, and the need to preserve ecological health. The complexity and prolonged timeframe of soil remediation significantly exceed those of water treatment, making it a critical frontier in environmental governance. Heavy metals (e.g., lead, arsenic) and persistent organic pollutants (e.g., polycyclic aromatic hydrocarbons, pesticides) exhibit low mobility and protracted natural degradation cycles. Conventional techniques like soil replacement and chemical washing are often cost-prohibitive (>$500 per ton) and can severely disrupt soil structure and microbial communities [51]. MNMs have emerged as promising innovative solutions due to their high reactivity and unique magnetic separability, which facilitates recovery after application. A substantial body of research demonstrates their efficacy in removing soil pollutants through mechanisms including adsorption/fixation, precipitation, catalytic oxidation (e.g., Fenton-like reactions), and microbial synergy.

### 4.1. Remediation of Heavy Metal Contamination

MNMs remediate heavy metal pollution primarily through adsorption fixation (via surface functional group coordination), chemical curing (forming stable mineral phases), and subsequent magnetic separation for recovery. Their high specific surface area and abundance of active sites enable effective binding of metal ions through ion exchange and electrostatic interactions. Crucially, MNMs can alter heavy metal speciation in soil, converting them from bioavailable forms (e.g., exchangeable, carbonate-bound) into more stable and less toxic residual forms, thereby reducing mobility and ecological risk.

#### 4.1.1. Research Advances

Several studies highlight this potential. Cui et al. [52] designed a biochar-supported magnetic nanomaterial (BMN)/sponge system that achieved an As (III) adsorption capacity of 16.23 mg/g and a magnetic recovery rate > 90% via Fe-O-As bonding. However, the material’s performance decreased by 30% in acidic soils (pH < 5) due to Fe^3+^ leaching, underscoring a key limitation. Liu et al. [53] developed a SiO_2_@Fe_3_O_4_@C-COOH solidifier that used carboxyl group coordination to increase the Pb solidification efficiency in contaminated soil to 85.1%. Nevertheless, a 12% increase in Pb leaching rates within three months post-remediation raised concerns about its long-term stability.

#### 4.1.2. Key Limitations for Heavy Metal Remediation

Despite the demonstrated efficacy of remediation in addressing these issues, certain limitations persist.

Firstly, there is a paucity of research on the long-term stability of these systems. The long-term stability of immobilized metals in dynamic soil environments is a major concern. Factors like wet-dry cycles, carbonation, and microbial activity can compromise curing agents, leading to potential remobilization and metal leaching. There is a critical paucity of long-term leaching risk studies under simulated field conditions and a lack of unified evaluation standards.

Secondly, MNMs have been observed to aggregate or dissolve in acidic or high-salinity soils, resulting in a loss of active sites. MNMs are susceptible to aggregation and dissolution in challenging soil chemistries. In acidic soils, MNMs can dissolve, releasing iron ions and losing active sites; acidity can also alter surface charge, prompting aggregation. In high-salinity soils, high ionic strength compresses the double electrical layer around particles, inducing aggregation and reducing dispersion and performance. Surface modifications can also be damaged, further degrading functionality.

### 4.2. Degradation of Organic Pollutants

MNMs degrade organic pollutants in soil through adsorption–catalysis synergy (e.g., Fenton-like reactions), enhancement of microbial electron transfer, and participation in redox cycles. Promising results have been demonstrated across various systems. Ma et al. [54] fabricated a Fe_3_O_4_-CuO@montmorillonite that catalyzed the ClO_2_ oxidation of anthracene with 96.2% efficiency. The system leveraged radical generation, electron transfer by Fe_3_O_4_, and adsorption by MMT. The catalyst was magnetically recovered and reused eight times with minimal activity loss or metal leaching (<0.1 mg L^−1^). Zhang et al. [55] established a synergistic system coupling Fe_3_O_4_-loaded biochar (Bio/MNs) with *Pseudomonas aeruginosa* bacteria, achieving a 98.6% removal rate of methylene blue under light irradiation, facilitated by enhanced electron transfer and radical generation. Zhao et al. [56] synthesized a magnetic MnFe_2_O_4_@soapberry-derived carbon (MnFe_2_O_4_@HL) composite material as a catalyst for a Fenton-like reaction, achieving a 92.3% degradation rate and 71.3% mineralization of tetracycline, attributed to a dual metal redox cycle accelerated by the carbon substrate.

However, the widespread application of MNMs for organic pollutant degradation continues to face several technical constraints that limit their practical implementation. A primary issue lies in the instability of surface modifications, which are often applied to mitigate particle agglomeration. In complex soil or aqueous systems, these functional coatings are prone to delamination or decomposition, resulting in reduced colloidal stability and loss of reactivity.

Furthermore, integrating MNMs into biological treatment systems presents additional difficulties. In particular, when MNMs are coupled with microbial communities to enhance degradation, challenges emerge in sustaining microbial activity and constructing efficient electron transfer pathways. High concentrations of MNMs may also disturb microbial ecology, leading to reduced biodiversity and impaired bioremediation functions.

Beyond performance hurdles, significant concerns regarding the ecological safety and adaptability of MNMs remain unresolved. The application of powdered nanomaterials introduces potential risks such as long-term persistence, bioaccumulation, and unintended toxicity toward non-target organisms. Therefore, ensuring material stability, environmental compatibility, and biosafety is crucial before MNMs can be reliably deployed in real-world remediation scenarios.

### 4.3. Challenges

In summary, MNMs show significant potential for addressing both heavy metal and organic pollution in soils through various advanced mechanisms. However, their transition from laboratory success to field-scale application is hindered by cross-cutting challenges. These include uncertainties regarding the long-term stability of immobilized pollutants or materials, the vulnerability of MNMs to soil chemistry (acidity, salinity), instability of surface modifications, potential toxicity of degradation products, and complex interactions with soil microbiomes. Furthermore, the biosafety and environmental risk of deploying nanomaterials in soils require meticulous scrutiny. Future research must focus on developing more robust and stable MNM designs, conducting long-term field trials under realistic conditions, and establishing comprehensive risk assessment frameworks to ensure the safe and effective application of MNM technology in soil remediation.

## 5. Advances in Biocompatibility Research

The pervasive utilization of magnetic nanomaterials (MNMs) in environmental remediation is increasingly scrutinized due to persistent concerns regarding their long-term ecological risks and biocompatibility. These concerns represent a significant impediment to their large-scale field implementation. The toxicological effects of MNMs are intricately linked to their intrinsic physicochemical properties—such as particle size, surface charge, and chemical composition—as well as extrinsic exposure conditions, including concentration and duration [57,58]. The fundamental challenges encompass metal ion leaching, ecological toxicity, instability of surface modifications, risks of migration and bioaccumulation, and overall diminished service life due to oxidation and deactivation.

### 5.1. Toxicity Mechanisms and Ecological Impacts

The toxic effects of unmodified or bare MNMs are triggered through multiple pathways, primarily driven by oxidative stress, which can lead to genetic damage and metabolic disruption.

#### 5.1.1. Cellular and Genetic Toxicity

Upon internalization, MNMs can release metal ions in acidic intracellular environments (e.g., lysosomes). For instance, Fe_3_O_4_ nanoparticles (<50 nm) release Fe^2+^/Fe^3+^ ions that catalyze intracellular H_2_O_2_ via the Fenton reaction, generating highly reactive hydroxyl radicals (•OH). This oxidative stress attacks cellular components, leading to lipid peroxidation, DNA damage, and ultimately apoptosis [59,60]. The genotoxic potential of MNMs is further illustrated by studies on aquatic organisms; for example, Zarria-Romero et al. [61] reported that a magnetized zeolite composite (MZ0) significantly downregulated the Glass gene (critical for eye development) in Daphnia magna, indicating interference with developmental pathways and reproductive toxicity, with larvae being the most vulnerable stage.

#### 5.1.2. Stability and Behavior in Biological Environments

The stability of MNMs is critical for their safety and efficacy. Organic coatings (e.g., polystyrene) can be decomposed by microorganisms in the environment, destabilizing the modification layer and exposing the magnetic core. This leads to agglomeration, which can reduce remediation efficiency by over 50% [62]. Furthermore, interaction with environmental biomolecules like proteins can cause charge reversal and reduced dispersibility, impairing targeted functionality [63].

#### 5.1.3. Impacts on Soil Microbial Communities and Ecosystem Functions

MNMs can significantly alter the structure and function of soil microbial communities, thereby disrupting key ecological processes. Predominant effects include reduced microbial diversity and imbalances in functional groups.

The addition of γ-Fe_2_O_3_ nanoparticles (0.1–1.0 mg/g soil) led to a 25–30% decline in specific fatty acids (16:1ω7c, 18:1ω7c) indicative of Gram-negative bacteria, suggesting oxidative damage to cell membranes [64]. Alloy MNMs containing cobalt or nickel can release toxic ions, reducing soil microbial diversity and decreasing organic matter decomposition efficiency by more than 30% [65]. MNMs profoundly disrupt nitrogen cycling [66]. Applications of biochar–nZVI composites (1% *w*/*w*) reduced bacterial diversity indices and slashed the abundance of nitrifying bacteria (Nitrosomonas) from 4.3% to 1.8%, impairing nitrification function [67]. Similarly, Fe_2_O_3_ nanoparticles were shown to reduce soil nitrogen mineralization efficiency by 29% and plant nitrogen recovery by 39% [68,69]. In a soil–sludge system, Fe_3_O_4_@SiO_2_ addition shifted microbial populations (Proteobacteria decreased, Firmicutes increased) and reduced total nitrogen removal efficiency by 18% [70]. These effects, observed within 4–12 weeks and dose-dependent, highlight the risk of weakening critical soil ecosystem functions.

#### 5.1.4. Knowledge Gaps in Toxicity Assessment

A critical limitation of existing toxicity data is its reliance on laboratory models involving single pollutants and short-term exposures (often <6 months). These conditions do not reflect the composite toxicity mechanisms of MNMs with co-existing pollutants in real environments [71]. The transgenerational genetic effects on higher organisms (e.g., mammals) remain largely unknown. A primary challenge is balancing magnetic performance with biosafety, as enhancing one often compromises the other (e.g., thicker coatings improve safety but reduce magnetic and catalytic efficiency).

### 5.2. Strategies for Enhancing MNM Biocompatibility

To mitigate the ecological risks of MNMs, several safety-by-design strategies have been developed, primarily focusing on surface engineering and the use of protective agents.

#### 5.2.1. Surface Modification

Surface modification stands as the most prevalent strategy for reducing the toxicity of magnetic nanomaterials (MNMs), with the choice of coating material critically determining their biocompatibility. Hydrophilic polymers, such as polyethylene glycol (PEG), dextran (DEX), and polyvinyl alcohol (PVA), are widely employed to enhance colloidal stability and minimize biointeractions [72]. A study demonstrated that human kidney cells (HEK293) were exposed to amino-functionalized core–shell magnetic nanoparticles (Fe_3_O_4_@mSiO_2_-NH_2_ NPs), both before and after Pb (II) adsorption. A significant enhancement in cell viability (up to 119.9%) was observed when treated with the pure NPs. In contrast, a dose-dependent decrease in viability (to 84.7%) was recorded after exposure to Pb^2+^-adsorbed NPs. However, at equal Pb^2+^ concentrations, the cytotoxicity induced by NP-adsorbed Pb^2+^ was found to be lower than that caused by free Pb^2+^ ions. This protective effect is attributed to the sequestration of Pb^2+^ by the functionalized NPs, which reduces its bioavailability and alters its intracellular distribution, thereby mitigating direct oxidative damage and acute toxicity [73].

Similarly, PEGylated silica–iron oxide nanocomposites of four sizes (~25, 45, 98, and 202 nm) exhibited low cytotoxicity in fibroblast and macrophage cell lines, with viability remaining high even at 1000 μg/mL in fibroblasts. However, at this high concentration, all sizes reduced macrophage viability, though significantly less than silica alone. A clear size-dependent inflammatory response was observed: only the smaller nanocomposites (20 and 40 nm) markedly stimulated IL-6 production in macrophages. This size effect is likely attributed to the higher cellular uptake efficiency and surface reactivity of smaller nanoparticles, which facilitates stronger interactions with immune cells and subsequent pro-inflammatory signaling. The superior biocompatibility compared to pure silica is primarily due to the protective PEG coating, which reduces direct cellular contact and mitigates toxicity, as evidenced by the significant polymer content (65% to 30% weight loss by TGA), providing steric stabilization and stealth properties [74].

Beyond polymer identity, the surface charge imposed by the coating is equally crucial. Inorganic coatings like silica shells (e.g., Fe_3_O_4_@SiO_2_) offer a robust physical barrier, effectively reducing metal ion leaching in acidic environments to less than 5% and suppressing reactive oxygen species (ROS) production by up to 70% [75]. In summary, while bare surfaces and coatings with aminosilane or cationic polymers (e.g., PEI) are considered high-risk due to their propensity for ROS generation and lysosomal damage, functionalization with carboxylic acids, PEG, DEX, or PVA substantially enhances biocompatibility and reduces genotoxicity. Nevertheless, the long-term epigenetic effects of even these safer coatings necessitate further thorough investigation.

#### 5.2.2. Synergism with Antioxidants

Combining MNMs with antioxidants provides a dual strategy: quenching harmful free radicals and modulating iron metabolism to reduce Fenton reaction substrates.

Fe_3_O_4_/reduced graphene oxide (rGO) nanocomposites loaded with natural antioxidants like resveratrol (RES) can donate hydrogen atoms from phenolic groups to neutralize free radicals, reducing oxidative DNA damage [76]. Encapsulation with edible biomaterials (e.g., soybean lecithin, hyaluronic acid, chitosan) improves biocompatibility through steric hindrance and charge modulation. Soybean lecithin coatings form micelles that isolate iron ions, limiting their participation in radical generation. A significant drawback of natural antioxidants is their potential to cause secondary pollution upon environmental release.

### 5.3. Summary and Future Perspectives

In summary, while significant progress has been made in understanding and mitigating MNM toxicity through surface engineering and antioxidant strategies, critical challenges persist. These include the potential loss of catalytic efficacy due to surface passivation, the immature state of controlled-release technologies for protective agents, and the issue of non-targeted adsorption in complex environmental media. Biological safety is the fundamental prerequisite for the environmental application of MNMs. Achieving a balance between high remediation performance and minimal ecological risk necessitates interdisciplinary collaboration and innovation in material design, advanced toxicity assessment under realistic long-term and multi-pollutant scenarios, and engineered applications that prioritize environmental compatibility.

## 6. Perspectives and Conclusions

### 6.1. Perspectives

MNMs represent a paradigm of cutting-edge materials in environmental remediation, demonstrating considerable potential for the removal of heavy metals, degradation of organic contaminants, and treatment of polluted soils, as consistently validated by laboratory studies. Their prominent advantages, including high specific surface area, superparamagnetism, and highly tunable surface chemistry, allow MNMs to serve not only as highly efficient adsorbents but also as catalytic platforms—enabling degradation via processes such as Fenton-like reactions—while permitting rapid recovery through external magnetic fields, thereby significantly reducing operational costs and material loss.

Despite these promising features, the transition to large-scale, real-world application remains constrained by several critical challenges. Key issues include material agglomeration, surface oxidation, instability under environmental conditions, uncertain long-term ecological fate, and non-standardized biosafety assessments [77]. Future research should prioritize the following aspects to bridge the gap between laboratory research and field implementation.

#### 6.1.1. Performance Optimization and Stability Enhancement

Future material design must focus on enhancing stability, selectivity, and reusability under realistic environmental conditions—such as fluctuating pH, temperature, and ionic strength—or in complex multi-pollutant systems. The development of “smart” MNMs with environmental responsiveness, self-healing properties, and magnetic-field guided functionality represents a promising direction for achieving targeted and efficient remediation [78].

#### 6.1.2. Comprehensive and Standardized Safety and Lifecycle Evaluation

A rigorous, internationally harmonized framework for assessing the biological and ecological safety of MNMs is essential. This should include long-term toxicity studies, multi-generational environmental impact assessments, and full life cycle analysis (LCA) to evaluate ecological footprints from synthesis to disposal.

#### 6.1.3. Interdisciplinary Integration and Technological Enablement

The convergence of materials science, environmental chemistry, nanotoxicology, and data science will be crucial. Artificial intelligence (AI) and machine learning (ML) can accelerate the development of next-generation MNMs through high-throughput screening and predictive modeling of material performance and environmental behavior. Meanwhile, green synthesis routes should be adopted to reduce the environmental burden associated with large-scale production.

### 6.2. Conclusions

In summary, recent years have witnessed significant advances in the development and application of MNMs for environmental remediation. Research has progressed from basic material synthesis to sophisticated functional design—such as surface modification with specific functional groups (e.g., amino, carboxyl, or metal oxide coatings) to enhance selectivity toward target pollutants like arsenic, copper, or mercury, and the construction of composite catalysts for efficient degradation of organic pollutants. Furthermore, applications have expanded from single-pollutant systems in idealized aqueous solutions to complex realistic media, including industrial wastewater and contaminated soils. Studies increasingly focus on competitive adsorption mechanisms, long-term stability, and environmental toxicity, paving the way for field-scale validation. Although challenges such as particle aggregation, surface oxidation, and environmental mobility remain, continuous innovation in material design—coupled with interdisciplinary collaboration—is steadily promoting the standardization and scaled application of MNMs. These efforts align closely with the overarching goals of green and sustainable environmental technology, positioning MNMs as a promising tool for achieving safe, efficient, and sustainable remediation solutions in the future.

## Figures and Tables

**Table 1:** Core properties, functions, and environmental applications of magnetic nanomaterials (MNMs). Summary based on data from Refs. [12,13,14,15,17,18,19].

Core Properties	Derived Functions	Application Targets	Representative Examples
High Surface Area	Provide abundant adsorption sites; High contaminant uptake capacity	Efficient adsorption of heavy metal ions (e.g., Pb^2+^, Cd^2+^, Cu^2+^)	Porous MNMs achieve adsorption capacities of 100–500 mg/g for metals.
Superparamagnetism	Enable rapid magnetic separation; Facilitate recovery and reuse; Prevent secondary release	Final separation step in all MNM-based applications	>99% recovery achieved within minutes under an external magnetic field.
Easy Functionalization	1. Introduce specific functional groups (-COOH, -NH_2_, -SH) 2. Enhance dispersion and stability 3. Improve selectivity for target contaminants	1. Targeted removal of specific pollutants (e.g., As, Cr (VI) 2. Improved performance in complex water matrices	Magnetic Hemicellulosic Composite Microspheres for Cu^2+^; Chitosan coatings for enhanced biocompatibility.
Catalytic Activity	Activate peroxydisulfate (PDS) or H_2_O_2_ to generate reactive oxygen species (ROS)	Catalytic degradation of organic pollutants (e.g., dyes, antibiotics, POPs)	MnFe_2_O_4_/PMS system degrades Bisphenol A; CuFe_2_O_4_ activates persulfate.

**Table 2:** Performance summary of selected magnetic nanomaterials (MNMs) for pollutant degradation.

Material (Configuration)	Target Pollutant	Optimal Conditions	Key Performance Metric	Primary Mechanism (s)	Ref.
Magnetic Hemicellulosic Composite Microspheres	Cu (II)	pH 5.0, initial Cu^2+^ concentration 80 mg/L	149.25 mg/g	Adsorption (hybrid magnetic composite microspheres)	[17]
Fe_3_O_4_-NPs (MAAC)	Cd (II)	150 mg/L, pH = 6	>90% removal 70 mg/g (Maximum adsorption capacity)	Adsorption	[32]
Fe_3_O_4_ @DA-DMSA (FDDMs)	Pb (II) Cu (II) Cd (II)		187.62 mg/g, 63.01 mg/g 49.46 mg/g (Maximum adsorption capacity)	Adsorption (Organic–inorganic hybrid)	[34]
Magnetic GO-Chitosan (Composite)	Hg (II)	-	339.82 mg/g (in 8 min)	Adsorption (Amidation)	[37]
Fe_3_O_4_@DABA	Congo red Eosin yellow	pH 3 25 °C.	259 mg/g 282 mg/g	Adsorption (Organic-functionalized)	[42]
Cu_0.5_Mn_0.5_Fe_2_O_4_ (Spinel Ferrite)	RhB	20 mg/L, Vis Light, 60 min	>99% degradation	Photo-Fenton	[43]
Fe_3_O_4_@CHC	RhB	25 °C, pH 3.0, 0.5 g/L catalyst, 10 mmol /L H_2_O_2_	90.2% degradation <0.3 mg/L (iron leakage)	Photo-Fenton (·OH generation)	[44]

## Data Availability

The original data presented in the study are included in the article; further inquiries can be directed to the corresponding author [N.L.].
